# Trends in urban/rural inequalities in cardiovascular risk bio-markers among Chinese adolescents in two decades of urbanisation: 1991–2011

**DOI:** 10.1186/s12939-018-0813-1

**Published:** 2018-07-11

**Authors:** Nan Zhang

**Affiliations:** Social Statistics, Cathie Marsh Institute, School of Social Sciences, The University of Manchester, Oxford Road, Manchester, M13 9PL UK

**Keywords:** Urbanisation, Neighbourhood effect, Inequality, Cardiovascular risk, China

## Abstract

**Background:**

China has seen rapid socio-economic changes and epidemiological transitions in the last few decades. Previous studies often fail to examine how wider macro-level forces contribute to changes in health inequality among its population in China. This study aims to examine urban/rural inequalities in cardiovascular (CVD) risks biomarkers among Chinese adolescents in two decades from 1991 to 2011 during the process of China’s rapid urbanisation.

**Methods:**

Data were drawn from a nationwide longitudinal dataset of the China Health and Nutrition Survey (CHNS) (Sweeps 1991, 1993, 1997, 2000, 2004, 2006, 2009 and 2011). Children aged between 12 and 18 years (Boys: *n* = 3472; Girl: *n* = 3155) were included. A dynamic urbanisation index was created for each community (village or neighbourhood) based on community-level data that can reveal the heterogeneity within and across places and capture dimensions of social, economic and physical characteristics of urban living over time and space. Linear multilevel modeling analyses (Level 1: Occasions; Level-2: Individuals; Level-3: Households; Level-4: Communities) were performed on outcomes of CVD risk biomarkers including anthropometric measures and blood pressure.

**Results:**

After adjustment for age, maternal education and household income per capita, cardiovascular (CVD) risk biomarkers increase among Chinese adolescents during 1991 to 2011. Urbanisation tends to have an independent and positive impact on body mass index (BMI) and waist circumference for boys but not for girls. Positive interaction effect between urbanisation index and survey years for waist circumference was observed for girls (0.005; 95% confidence interval [CI], 0.002, 0.007; *p* < 0.01): time trends become greater when areas become more urbanized. For blood pressure, when areas become more urbanized the trends for boys become decreased (− 0.005; 95% CI, − 0.009, − 0.002; *p* < 0.01 for systolic blood pressure and − 0.003; 95% CI, − 0.006, − 0.001; *p* < 0.05 for diastolic blood pressure).

**Conclusion:**

Chinese adolescents are experiencing an upward trend of cardiovascular (CVD) risks in last two decades. Its rapid urbanisation appears to further increase urban/rural inequalities in CVD risks, especially for boys from less urbanised areas and girls from more urbanised areas, which may contribute to the development of cardiovascular disease (CVD) in adulthood. It is relevant to inform policy making process to target specific vulnerable groups. Given China’s urbanisation is strongly influenced by the state, there is a possibility for policy intervention to reduce inequality during the process of China’s planned urbanisation.

## Background

Urban living affects children’s nutrition and growth, which determine their survival, cognitive development and lifelong health. It has been well documented that urban children tend to fare better than their rural counterparts [1–3]. Urban environments have been linked to a range of human health issues. Three approaches have been proposed regarding research on urban health [4]. Urban health penalty and urban sprawl focus on the health problems of cities [5]. The urban penalty approach posits that cities concentrate poor people and expose them to unhealthy physical and social environments. Urban sprawl focuses on the adverse health and environmental effects of urban growth into outlying area. The urban penalty and urban sprawl approaches concentrate on negative aspects of health, and fail to consider the strengths within metropolitan areas. The third approach, ‘urban health advantage’, considers the special resources and protective effect of cities and only focuses on the positive aspects of urban living. All three perspectives are relatively static, describing health within a particular stage of urban development rather than exploring the changing processes between and within cities and their surrounding areas [5].

Urbanisation is a complex phenomenon, associated with a large array of changes in environmental and lifestyle factors that may affect individual health and wellbeing. As the pace of urbanisation accelerates, new challenges arise to characterise these environments, and to understand their positive and negative implications for health [6]. However, attributing diseases to specific environmental influences has proved elusive [7]. Factors beyond including the social and physical environment (not just individual risk factors) are the primary determinants of health and wellbeing of people [4]*.* The influence of urbanisation on health can be mixed. On the one hand, there are the benefits of ready access to healthcare, sanitation, and secure nutrition, whilst on the other there are the evils of overcrowding, pollution, social deprivation, crime, and stress-related illness.

China is a good study case to examine the influence of urbanisation on population health given the size of its population, the special conditions of market socialism and its institutional capacity to manage the rural-urban transformation. China will reach the same levels of urbanisation much quicker and the number of people involved in this shift will be much larger compared to other countries [8]. Unlike Western and former socialist countries, China’s rapid urbanisation is not merely driven by spontaneous rural-urban migration, but also partly planned by the government that has maintained an urban bias in favour of urban residents to preserve regime stability [9, 10]. The process of China’s rapid urbanisation may cause the unequal distributions of socio-economic resources and life chances among its population, thereby contributing to rural-urban health inequalities [11].

Alongside its rapid socio-economic change, China has also experienced an epidemiological transition shifting from infectious to chronic diseases in a much shorter time frame than many other countries [12]. Lessons learned from epidemiological studies mostly carried out in Europe and the US may not be directly transferable to China [7]. Children and adolescents in China are becoming increasingly overweight and obese over the past decades [13–16], particularly among higher socioeconomic children [17]. Liang et al. [14] analysed the CHNS data and have found that blood pressure levels and prevalence of hypertension increased dramatically among Chinese children and adolescents aged 6–17 years from 1991 to 2004. Obesity itself has been shown to be strongly associated with the onset of hypertension [18, 19]. Children with higher blood pressure are more likely to become hypertensive adults [20–22] and further increase the risk of cardiovascular diseases [23]. It has been suggested that targeting at childhood or early years of life has the potential to reduce health inequalities within one generation [24]. This study focused on adolescents because they are more likely to be affected by neighbourhoods directly than younger children for whom neighbourhood effects may be mediated through family processes [25–27], since most of their time is spent at home and their interactions with larger social and physical contexts are determined by and largely experienced through their parents/caregivers.

Most studies that examined the health consequences of urbanisation employ a dichotomous urban-rural classification of individuals living in either urban or rural environments. This approach ignores the heterogeneity of experiences within and across urban and rural situations, and fails to consider the specific aspects of local environments that are closely related to health outcomes [6]. This is particularly relevant given substantial regional variations in economic development and public resources across China. Another pitfall of employing urban-rural dichotomy is its inability to detect changes in urbanisation over time [28]. This makes it impossible to examine temporal trends and patterns of urbanisation on health outcomes among adolescents in China undergoing rapid urbanisation in the last few decades.

Despite well documented rural-urban inequalities in child health in China, there are few attempts to examine how these relationships have changed during the process of China’s urbanisation. Previous research tends to rely on a cross-sectional study design and failed to examine the dynamics in child health in a rapidly urbanizing society. This study will ask three questions:

1. What is the trend in cardiovascular (CVD) risk biomarkers among Chinese adolescents in the last two decades?
2. Does urbanisation has an independent (of family socioeconomic status) impact on cardiovascular risk biomarkers among Chinese adolescents?
3. Do the trends of cardiovascular risk biomarkers change according to the degrees of urbanisation?

## Methods

### Study design and participants

Data were drawn from the CHNS, an on-going open-cohort study that employs a multistage, random-clustered sampling process to draw a sample of about 4400 households with a total of about 19,000 participants (children and their family members available) from over 200 communities or neighbourhoods in nine provinces, with the first round conducted in 1989. The CHNS covers nine provinces that vary substantially in geography, economic development, public resources and health indicators. The design, sampling and response rates are reported elsewhere [29]. The sample began with eight provinces (Liaoning, Jiangsu, Shandong, Henan, Hubei, Hunan, Guangxi and Guizhou) and added a ninth, Heilongjiang, in 1997 and three autonomous cities, Beijing, Shanghai, and Chongqing, in 2011. We used data from eight waves of the CHNS, collected in 1991, 1993, 1997, 2000, 2004, 2006, 2009, and 2011. We included nine provinces and excluded three autonomous cities in 2011 from the analysis. There were 11,583 observations of children with 6055 boys and 5528 girls. After excluding those with missing data on key variables, 6627 observations of children (3472 boys and 3155 girls) were included in the analyses.

### Outcomes

A variety of objective biomarkers were examined including Body Mass Index (BMI), waist circumferences (WC), systolic blood pressure and diastolic blood pressure [30].

The CHNS recorded height and weight for each individual within the household, as measured by health professionals. Height was measured without shoes to the nearest 0.1 cm using a portable stadiometer; weight was measured without shoes and in light clothing to the nearest 0.1 kg on a calibrated beam balance. BMI (kg/m^2^) was calculated as weight (kg) divided by height squared (m^2^). waist circumference (cm) was measured using an on-elastic tape at a point midway between the lowest rib margin and the iliac crest in a horizontal plane [31]. Waist circumference is a relatively simple and convenient measure and can be readily used to estimate the accumulation of abdominal fat among children due to their rapid growth and development [32].

Systolic and diastolic blood pressure were measured on the right arm, using mercury sphygmomanometers with appropriate cuff sizes [33]. Measures were collected in triplicate after a 10 min seated rest and the mean of the three measurements was used in analyses.

### Exposure

It is increasingly recognised that a simple urban-rural dichotomy is an over simplification which cannot adequately detect heterogeneity in living and health conditions within urban and rural areas at different stages of urbanisation [6, 28, 34]. In this study, a dynamic measure is urbanisation score, which was based on in-depth community contextual measures that captures major dimensions of modernization across all 288 communities currently in the CHNS sample. The standardized, validated measure captures the changes in 12 dimensions through exploratory factor analysis: population density, economic activity, traditional markets, modern markets, transportation infrastructure, sanitation, communications, housing, education, diversity, health infrastructure, and social services [35]. Each is based on numerous measures relevant to each dimension [36] and can distinguish features of urban-rural places [35]. This index is a reliable and valid scale with a Cronbach’s alpha higher than 0.85 [35]. This study treats urbanisation as a process of accumulation of urban elements and uses a composite urbanisation index that sees urbanisation as a spectrum, which is more effective to reveal the heterogeneity within and across places.

### Covariates

Covariates included age (centred at age 12 years), quadratic term of age, gender, maternal education (completed years of formal education in regular school), log transformation of per capita household income (inflated to 2011) and survey years (1991, 1993, 1997, 2000, 2004, 2006, 2009 and 2011).

### Statistical analysis

Since the CHNS is hierarchically structured with repeated measures nested within participants clustered within households and further nested within communities (villages or neighbourhoods), multilevel models were fitted with repeated measurements at level-1, participants at level-2 and communities at level-3 to correct for non-independence of observations due to geographic clustering and repeated observations of individuals [37]. All analyses were constructed within Stata/SE 13 [38]. The log likelihood ratio test was used to determine the preferred model among nested models.

We first fitted a quadratic function of age and survey years in Model 1 to examine if there are any significant trends in outcomes. Then we further adjusted for urbanisation index to see if urbanisation has an independent impact on outcomes (Model 2). In order to examine if the impact changes over time, we further adjusted for the interaction between survey year and urbanisation index (Model 3). All models were adjusted for family socioeconomic circumstances in terms of maternal education and household income per capita to remove potentially confounding effects. All analyses were stratified by gender given the specific socio-cultural context of China where boys tend to be more valued and given more resources [39–41].

For the simplicity of interpretation, we presented plots of the time trends on outcomes (only significant interactions between urbanisation index and survey years were shown). We illustrated the predicted relationships between outcomes and survey years stratified by two levels of urbanisation in terms of the most urbanised areas (the top 20th percentile) and the least urbanised areas (the bottom 20th percentile). Those plots can offer an overall picture of trends in urban/rural inequalities in bio-makers among Chinese adolescents over two decades.

## Results

Tables 1 summarizes characteristics of adolescents aged 12–18 by gender and survey years, the CHNS 1991–2011. The age distributions of study participants did not show significant differences across survey years. Family socioeconomic circumstances measured by maternal education and per capita household income appear to improve over 20 years. With regards to anthropometric measures (height, weight, BMI and WC), adolescents became taller and heavier over time. For systolic and diastolic blood pressure measurements, trends do not show a consistent pattern. Boys tend to have higher blood pressure than girls over last two decades.

**Table 1:** General characteristics of adolescents aged 12–18 by gender over time, the CHNS 1991 to 2011^ab^

Variables	1991	1993	1997	2000	2004	2006	2009	2011
Boys	(*n* = 892)	(*n* = 796)	(*n* = 801)	(*n* = 983)	(*n* = 811)	(*n* = 724)	(*n* = 546)	(*n* = 502)
Age (Years)	15.15 (1.75)	14.93 (1.75)	14.98 (1.72)	14.71 (1.72)	15.37 (1.62)	15.35 (1.73)	15.09 (1.76)	14.98 (1.73)
Index	42.07 (15.55)	44.19 (15.43)	49.37 (16.91)	54.59 (16.97)	57.24 (19.60)	59.29 (19.62)	62.61 (18.81)	64.78 (18.85)
Maternal education (years)	3.46 (3.68)	4.16 (3.79)	5.73 (4.03)	6.97 (3.82)	7.30 (3.54)	7.53 (3.67)	7.79 (3.35)	8.25 (3.07)
Log per capita household income	7.64 (0.87)	7.63 (1.13)	7.91 (1.15)	8.08 (1.17)	8.11 (1.57)	8.27 (1.57)	8.68 (1.39)	8.74 (1.72)
Height (cm)	155.66 (11.64)	155.95 (11.80)	156.59 (11.73)	157.39 (11.41)	161.87 (10.30)	161.15 (11.89)	161.43 (10.65)	161.36 (11.43)
Weight (kg)	45.24 (10.63)	45.45 (10.39)	46.21 (10.72)	46.34 (11.23)	50.65 (10.98)	49.77 (11.41)	50.16 (11.43)	51.25 (13.26)
BMI (kg/m^2^)	18.45 (2.45)	18.44 (2.37)	18.60 (2.55)	18.48 (2.68)	19.16 (2.75)	19.04 (2.73)	19.09 (3.31)	19.44 (3.69)
Waist circumference (cm)	–	66.28 (7.35)	66.55 (7.57)	66.88 (8.02)	69.42 (9.10)	69.75 (9.40)	69.01 (9.12)	70.34 (12.23)
Systolic blood pressure (mm Hg)	102.39 (13.34)	102.11 (12.66)	103.31 (12.25)	104.11 (12.85)	107.20 (12.52)	104.85 (11.86)	104.82 (11.91)	105.57 (12.45)
Diastolic blood pressure (mm Hg)	66.49 (9.77)	66.63 (9.83)	67.38 (9.19)	67.65 (9.40)	69.94 (9.02)	68.78 (9.35)	69.12 (9.03)	69.86 (8.95)
Girls	(*n* = 833)	(*n* = 776)	(*n* = 737)	(*n* = 886)	(*n* = 726)	(*n* = 638)	(*n* = 487)	(*n* = 445)
Age (Years)	15.04 (1.74)	15.02 (1.74)	14.96 (1.74)	14.81 (1.72)	15.17 (1.67)	15.41 (1.64)	14.99 (1.81)	15.11 (1.64)
Index	42.43 (15.26)	43.80 (15.19)	50.40 (17.62)	55.86 (17.13)	57.10 (19.48)	59.57 (18.71)	63.88 (18.83)	65.47 (18.75)
Maternal education (years)	3.54 (3.60)	4.23 (3.73)	5.50 (4.14)	6.78 (3.93)	7.29 (3.58)	7.47 (3.77)	7.69 (3.66)	7.91 (3.49)
Log per capita household income	7.53 (1.06)	7.56 (1.29)	7.88 (1.12)	8.00 (1.31)	8.07 (1.54)	8.30 (1.41)	8.63 (1.31)	8.62 (1.68)
Height (cm)	151.19 (7.92)	151.94 (7.78)	152.59 (8.11)	153.62 (7.84)	154.96 (8.06)	155.88 (7.76)	155.08 (8.62)	155.61 (8.37)
Weight (kg)	43.40 (8.04)	43.21 (7.91)	43.99 (8.51)	44.35 (8.63)	45.78 (8.66)	45.84 (8.18)	46.00 (9.77)	47.49 (10.22)
BMI (kg/m^2^)	18.89 (2.62)	18.61 (2.43)	18.75 (2.56)	18.65 (2.74)	18.96 (2.64)	18.80 (2.65)	19.00 (3.00)	19.49 (3.27)
Waist circumference (cm)	–	65.37 (6.97)	65.29 (7.01)	65.13 (7.31)	66.00 (7.67)	66.09 (6.87)	66.14 (7.84)	68.99 (9.38)
Systolic blood pressure (mm Hg)	100.49 (12.14)	100.78 (10.97)	102.35 (11.92)	102.42 (11.43)	104.53 (10.43)	101.59 (10.33)	102.74 (10.78)	103.61 (9.90)
Diastolic blood pressure (mm Hg)	65.82 (8.91)	66.36 (8.30)	67.61 (9.39)	67.05 (8.75)	68.83 (8.46)	67.36 (7.98)	68.38 (8.66)	67.36 (8.17)

^a^Mean (sd) were presented; sd, standard deviations

^b^*CHNS* China Health and Nutrition Survey

Tables 2 and 3 present trends in anthropometric measures including BMI and waist circumference among adolescents aged 12–18 years in China. Positive trends were observed in BMI for boys and girls (Model 1, Table 2). Positive trends persisted after adjusting for urbanisation index (Model 2, Table 2) for both genders. There was no significant interaction effect between urbanisation index and survey years on BMI. With regards to waist circumference, a positive trend was observed for both genders before and after controlling for urbanisation index (Model 1 & 2, Table 3). A significant interaction (between urbanisation index and survey years) was observed for girls: the positive trend became greater when urbanisation index increased (Model 3, Table 3).

**Table 2:** Trends in the influence of urbanisation on body mass index (BMI) of adolescents aged 12–18 years, the CHNS 1991 to 2011^ab^

	Model 1		Model 2		Model 3	
	Coefficient	95%CI	Coefficient	95%CI	Coefficient	95%CI
Boy						
Year	0.039^***^	(0.022 0.055)	0.029^**^	(0.011, 0.047)	0.056^*^	(0.012, 0.100)
Index			0.011^**^	(0.004, 0.018)	1.008	(− 0.479, 2.495)
Year^*^Index					−0.0005	(− 0.001, 0.0002)
Girl						
Year	0.023^**^	(0.006, 0.039)	0.027^***^	(0.009, 0.046)	−0.008	(− 0.053, 0.038)
Index			−0.005	(− 0.013, 0.002)	−1.295	(−2.820, 0.230)
Year^*^Index					0.0006	(− 0.0001, 0.001)

^*^P < 0.05; ^**^ *P* < 0.01; ^***^ *P* < 0.001

^a^*CHNS* China Health and Nutrition Survey, *CI* confidence interval

^b^All models adjusted for age (centred on 12 years), quadratic term of age, maternal education (completed years of formal education in regular school) and log transformation of per capita household income

**Table 3:** Trends in the influence of urbanisation on waist circumference of adolescents aged 12–18 years, the CHNS 1991 to 2011^ab^

	Model 1		Model 2		Model 3	
	Coefficient	95%CI	Coefficient	95%CI	Coefficient	95%CI
Boy						
Year	0.267^***^	(0.204, 0.330)	0.240^***^	(0.173, 0.308)	0.266^**^	(0.081, 0.451)
Index			0.029^*^	(0.004, 0.055)	0.941	(−5.147, 7.029)
Year^*^Index					−0.0005	(−0.003, 0.003)
Girl						
Year	0.177^***^	(0.119, 0.236)	0.175^***^	(0.113, 0.237)	−0.081	(− 0.253, 0.091)
Index			0.005	(−0.018, 0.028)	−9.024^**^	(−14.656, −3.391)
Year^*^Index					0.005^**^	(0.002, 0.007)

^*^P < 0.05; ^**^ *P* < 0.01; ^***^ *P* < 0.001

^a^*CHNS* China Health and Nutrition Survey, *CI* confidence interval

^b^All models adjusted for age (centred on 12 years), quadratic term of age, maternal education (completed years of formal education in regular school) and log transformation of per capita household income

**Fig. 1:**
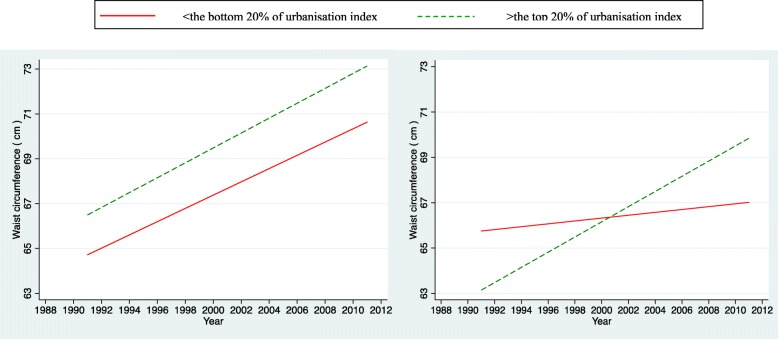
Trends in the influence of urbanisation on waist circumference of adolescent boys (left) and girls (right) aged 12–18 years in China

Figure 1 plots the time trends of waist circumference for the most urbanised and the least urbanised areas for boys and girls. We found significant and positive interaction effects between survey years and urbanisation index among girls, but not for boys. The difference between the most urbanized and the least urbanized areas remained unchanged for boys over two decades (interaction not significant). This may indicate the absence of urban-rural inequality in waist circumference among Chinese adolescent boys during China’s rapid urbanisation. The pattern appears more complex for girls. The most urbanized areas tend to have lower waist circumference than the least urbanized by 2004 and the gap continues to narrow. From 2004 onwards, a reversed pattern was observed among girls whereas girls from the most urbanized areas tend to be affected more in the association between waist circumference and survey years than those from the least urbanized areas, and the disparity further widened.

Tables 4 and 5 present trends in blood pressure among adolescents aged between 12 and 18 years in China. Positive trends were observed for both boys and girls (Model 1) and persisted after taking into account of urbanisation index (Model 2). The positive trends appear to decline when areas became more urbanized (Model 3). This was only observed for boys but not for girls.

**Table 4:** Trends in the influence of urbanisation on systolic blood pressure of adolescents aged 12–18 years, the CHNS 1991 to 2011^ab^

	Model 1		Model 2		Model 3	
	Coefficient	95%CI	Coefficient	95%CI	Coefficient	95%CI
Boy						
Year	0.192^***^	(0.116, 0.269)	0.186^***^	(0.104, 0.269)	0.480^***^	(0.268, 0.693)
Index			0.006	(−0.029, 0.042)	10.774^**^	(3.614, 17.934)
Year^*^Index					−0.005^**^	(−0.009, − 0.002)
Girl						
Year	0.228^***^	(0.156, 0.299)	0.255^***^	(0.177, 0.333)	0.337^***^	(0.136, 0.538)
Index			−0.030	(−0.063, 0.003)	2.957	(−3.817, 9.731)
Year^*^Index					−0.001	(− 0.005, 0.002)

^*^P < 0.05; ^**^ *P* < 0.01; ^***^ *P* < 0.001

^a^*CHNS* China Health and Nutrition Survey, *CI* confidence interval

^b^All models adjusted for age (centred on 12 years), quadratic term of age, maternal education (completed years of formal education in regular school) and log transformation of per capita household income

**Table 5:** Trends in the influence of urbanisation on diastolic blood pressure of adolescents aged 12–18 years, the CHNS 1991 to 2011^ab^

	Model 1		Model 2		Model 3	
	Coefficient	95%CI	Coefficient	95%CI	Coefficient	95%CI
Boy						
Year	0.144^***^	(0.087, 0.201)	0.134^***^	(0.072, 0.196)	0.303^***^	(0.147, 0.460)
Index			0.010	(−0.016, 0.035)	6.226^*^	(0.943, 11.509)
Year^*^Index					−0.003^*^	(− 0.006, − 0.001)
Girl						
Year	0.163^***^	(0.108, 0.217)	0.165^***^	(0.106, 0.223)	0.193^***^	(0.042, 0.344)
Index			−0.002	(−0.026, 0.022)	1.019	(−4.056, 6.095)
Year^*^Index					−0.0005	(−0.003, 0.002)

^*^P < 0.05; ^**^ *P* < 0.01; ^***^ *P* < 0.001

^a^*CHNS* China Health and Nutrition Survey, *CI* confidence interval

^b^All models adjusted for age (centred on 12 years), quadratic term of age, maternal education (completed years of formal education in regular school) and log transformation of per capita household income

**Fig. 2:**
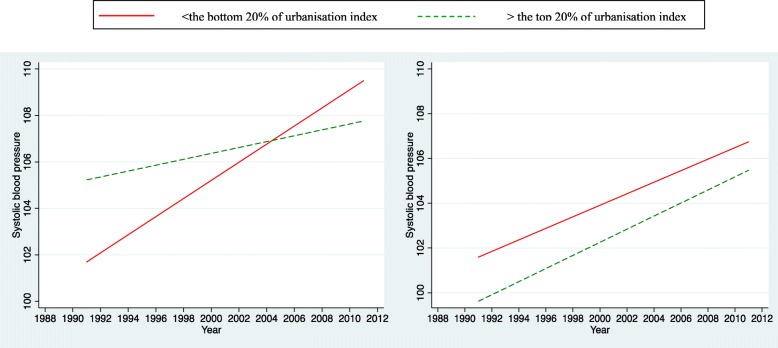
Trends in the influence of urbanisation on systolic blood pressure of adolescent boys (left) and girls (right) aged 12–18 years in China

**Fig. 3:**
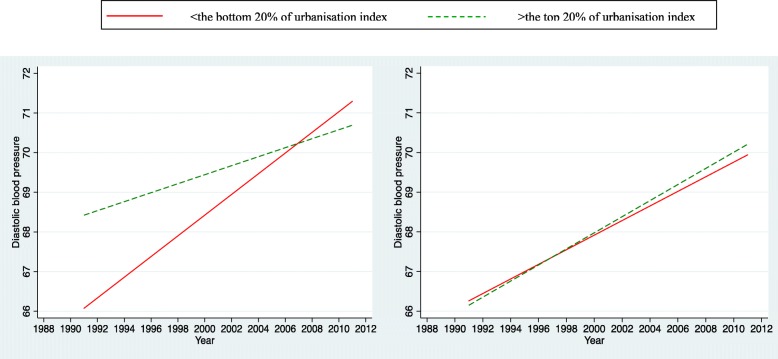
Trends in the influence of urbanisation on diastolic blood pressure of adolescent boys (left) and girls (right) aged 12–18 years in China

Figures 2 and 3 illustrate the time trends of systolic and diastolic blood pressure for both the most urbanized and the least urbanized areas for boys and girls separately. An upward trend was observed for both genders regardless of levels of urbanisation. For boys, those from the most urbanized areas appear to have higher systolic blood pressure than those from the least urbanized. However the gap continued to converge till 2004; and after that the gap widened with the least urbanized areas overriding or surpassing the most urbanized. For girls, those from the least urbanized areas appear to have higher systolic blood pressure than those from the most urbanized areas. This pattern persisted and remained constant over two decades (interaction not significant). A similar pattern for diastolic blood pressure was observed for boys (Fig. 3).

## Discussion

Our study provides an overall picture of time trends of biomarkers among Chinese adolescents and how it changes with urbanisation over last two decades. It has drawn on a nationwide longitudinal dataset–the CHNS to examine trends in related biomarkers among adolescents aged 12–18 years in China by considering an important wider contextual factor–urbanisation. Chinese adolescents are experiencing an upward trend of cardiovascular risks in the last two decades. Its rapid urbanisation appears to further increase the risk, especially for boys from less urbanised areas and girls from more urbanised areas, which may contribute to the development of cardiovascular disease in adulthood. There are at least two strengths of this study compared to previous evidence. First, the use of multilevel modeling techniques in this study allows for disentangling effects due to individual, family and neighborhood characteristics, which is methodologically more robust. Therefore it can provide an opportunity for deeper insights into how features of urban living may impact health [4]. Another advantage of this study is, unlike previous studies mostly drawing on the crude classification of rural/urban dichotomy that is prone to misclassification error, our urbanisation measure has encompassed dimensions beyond population size and density. It has captured some important dimensions of social, economic and physical characteristics of urban living over time and space [36].

### Comparison with previous studies

We found positive trends in all cardiovascular risk biomarkers (BMI, waist circumference, systolic blood pressure and diastolic blood pressure) among Chinese adolescents over two decades after adjustment for relevant confounders at individual and household levels. This is consistent with previous studies that found increased prevalence of obesity (and overweight) [13, 15, 16] and hypertension among Chinese children [14, 42].

Our study has shown that urbanisation has an independent and positive impact on BMI and waist circumferences. This is consistent with existing evidence that the obesity rate in children and adolescents increases with the degree of urbanisation in China [43, 44]*.* Increased BMI and waist circumference indicate increased risk of overall obesity and central obesity, respectively. One potential explanation for its positive relationships with urbanisation index may be due to the fact of potentially negative influence of urban living regarding unhealthy diets and lifestyles (physical inactivity and increased access to high-fat and energy-dense diets), as well as exposure to obesogenic environment [45–47]. These changes have already shifted to poor and rural areas [48]. We observed positive influences of urbanisation on BMI and waist circumference for boys only. This may suggest that living in more urbanized areas can be more detrimental for boys than for girls. One possible explanation may be due to the socio-cultural norm of ‘son preference’ whereas sons are more culturally valued and tend to be allocated more resources than daughters [39–41]. Another possible explanation may be that adolescent boys are more prone to develop risky cardiovascular health behaviors such as poorer dietary habits, less physical activity, and higher odds of smoking that can be associated with poor neighborhood contexts [49]*.*

We found positive trends in blood pressure for Chinese adolescents from 1991 to 2011. Previous study found an upward trend in blood pressure among children aged 6–17 years from 1991 to 2004 [14]. They did not look at waves from 2004 onwards although they were already available by the time of publication. We found significant interaction effects between urbanisation index and survey years on blood pressure for boys only but not for girls: the positive trends tend to decline when areas become more urbanized. Our findings suggest that the gap between the most urbanized and the least urbanized areas tends to first narrow then widen whereas adolescents from the least urbanized areas tend to have higher blood pressure than those from the most urbanized areas. To our best knowledge, we have not found relevant studies that have examined how trends in blood pressure among Chinese adolescents change according to the degrees of urbanisation. However, there is evidence suggesting that Chinese population have the relatively higher cardio metabolic risk at relatively lower BMI and younger ages [50] and higher insulin resistance relative to Western population despite the same level of BMI [51], which may further complicate the disease profile in China.

### Limitations

Several methodological limitations warrant cautious interpretation of our findings. The first concerns our measure of urbanisation. There is no standard definition for the classification of urban environment in less developed countries [52]. Urbanisation is a complex phenomenon that involves social, economic and environmental factors that are exogenous to the individual [4, 53]. Although our classification of urbanisation has captured some important dimensions of social, economic and physical characteristics of urban living over time and space [36]. There are still other facets in relation to urban environment that matter to adolescents’ health and wellbeing, for example, pollution [54, 55] and social cohesion [56]. Future studies may benefit from collecting a wide array of data on different dimensions of urbanisation in order to disentangle causal links between urbanisation and outcomes.

Second, this study aims to examine trends of biomarkers among adolescents in China and how it changes over the degrees of urbanisation. We have attempted to adjust for relevant confounders such as child attributes and family socioeconomic circumstances. There are, however, some key confounders that should be considered but they were not available from the secondary dataset – the CHNS, such as birthweight, physical activities and food choices within neighbourhoods which are closely related to adolescents’ health outcomes in China. Despite dietary and lifestyle changes, urbanisation and urban expansion also result in urban environmental changes, which can cause additional burden on population health profiles. For example, environmental risk factors, in particular air and water pollution, are one of the major contributors to morbidity and mortality in China [47]. As the country urbanises, the degree of transition to western-style diets and sedentary lifestyles, the trend in outdoor pollution, and progress in prevention of accidents are important factors that will affect the future health of China’s population [54].

Unfortunately, we were unable to distinguish the different mechanisms through which urbanisation affects children’s health. The pathways through which urbanisation affects children’s health and development can be complex and multi-factorial. Freudenberg et al. [5] proposed an alternative and more comprehensive approach that focuses on urban living conditions. A more comprehensive framework for urban health should incorporate and integrate the penalty and sprawl concepts as well as consider other features of living in cities that influence health. Dimensions of urban living conditions include the physical environment, the social environment, health and social services systems, and the characteristics of urban populations (i.e., behaviors, beliefs, and demographics) [5]. Future studies may benefit further from exploring and identifying modifiable risk factors and pathways that can prevent avoidable health inequalities.

And finally, one major limitation with this, and any longitudinal dataset, is missing data and attrition. In the CHNS, older children may not take part in later surveys, and school children who were in boarding schools, and who subsequently entered colleges and universities, may miss certain rounds of survey. Also, children may themselves migrate when aged above 16 years old [29]. Sample attrition and missing data could thus lead to biased results. However, we were unable to distinguish the exact direction of bias that was introduced by sample attrition and missingness in our estimates.

## Conclusion

Chinese adolescents are experiencing an upward trend of cardiovascular risks in last two decades. Its rapid urbanisation appears to further increase urban/rural inequalities in cardiovascular risks, especially for boys from less urbanised areas and girls from more urbanised areas, which may contribute to the development of cardiovascular disease in adulthood. Findings may provide a sound evidence basis to inform decision-making in public health and social welfare in relation to tackling health and welfare inequalities among children in China and potentially other developing countries that are undergoing similar socio-economic change and associated nutritional and epidemiological transitions. Unlike the detrimental influence of urbanisation and migration in other developing countries which often resulted in shantytowns and slums causing public health and other subsequent social problems, China’s ‘planned’ urbanisation may be beneficial to its population by providing opportunities for better health infrastructure, access to healthcare services, and essential services. There is a possibility for policy intervention to reduce inequality during the process of China’s planned urbanisation.

## Abbreviations

BMI: Body mass index
CHNS: China Health and Nutrition Survey
CI: Confidence interval
sd: Standard deviation
WC: Waist circumference
